# A Sensitive Whole Blood Assay Detects Antigen-Stimulated Cytokine Release From CD4+ T Cells and Facilitates Immunomonitoring in a Phase 2 Clinical Trial of Nexvax2 in Coeliac Disease

**DOI:** 10.3389/fimmu.2021.661622

**Published:** 2021-05-19

**Authors:** Melinda Y. Hardy, Gautam Goel, Amy K. Russell, Swee Lin G. Chen Yi Mei, Gregor J. E. Brown, Suyue Wang, Evan Szymczak, Ruan Zhang, Kaela E. Goldstein, Kristin M. Neff, Leslie J. Williams, Kenneth E. Truitt, John L. Dzuris, Jason A. Tye-Din, Robert P. Anderson

**Affiliations:** ^1^ Immunology Division, The Walter and Eliza Hall Institute of Medical Research, Parkville, VIC, Australia; ^2^ Department of Medical Biology, University of Melbourne, Parkville, VIC, Australia; ^3^ ImmusanT, Inc., Cambridge, MA, United States; ^4^ Department of Gastroenterology, Box Hill Hospital, Box Hill, VIC, Australia; ^5^ Department of Gastroenterology, Alfred Hospital, Prahran, VIC, Australia; ^6^ Department of Gastroenterology, The Royal Melbourne Hospital, Parkville, VIC, Australia

**Keywords:** coeliac disease, T cells, cytokines, cytokine release assay, IL-2, diagnosis

## Abstract

Improved blood tests assessing the functional status of rare gluten-specific CD4+ T cells are needed to effectively monitor experimental therapies for coeliac disease (CD). Our aim was to develop a simple, but highly sensitive cytokine release assay (CRA) for gluten-specific CD4+ T cells that did not require patients to undergo a prior gluten challenge, and would be practical in large, multi-centre clinical trials. We developed an enhanced CRA and used it in a phase 2 clinical trial (“RESET CeD”) of Nexvax2, a peptide-based immunotherapy for CD. Two participants with treated CD were assessed in a pilot study prior to and six days after a 3-day gluten challenge. Dye-dilution proliferation in peripheral blood mononuclear cells (PBMC) was assessed, and IL-2, IFN-γ and IL-10 were measured by multiplex electrochemiluminescence immunoassay (ECL) after 24-hour gluten-peptide stimulation of whole blood or matched PBMC. Subsequently, gluten-specific CD4+ T cells in blood were assessed in a subgroup of the RESET CeD Study participants who received Nexvax2 (maintenance dose 900 μg, n = 12) or placebo (n = 9). The pilot study showed that gluten peptides induced IL-2, IFN-γ and IL-10 release from PBMCs attributable to CD4+ T cells, but the PBMC CRA was substantially less sensitive than whole blood CRA. Only modest gluten peptide-stimulated IL-2 release could be detected without prior gluten challenge using PBMC. In contrast, whole blood CRA enabled detection of IL-2 and IFN-γ before and after gluten challenge. IL-2 and IFN-γ release in whole blood required more than 6 hours incubation. Delay in whole blood incubation of more than three hours from collection substantially reduced antigen-stimulated IL-2 and IFN-γ secretion. Nexvax2, but not placebo treatment in the RESET CeD Study was associated with significant reductions in gluten peptide-stimulated whole blood IL-2 and IFN-γ release, and CD4+ T cell proliferation. We conclude that using fresh whole blood instead of PBMC substantially enhances cytokine secretion stimulated by gluten peptides, and enables assessment of rare gluten-specific CD4+ T cells without requiring CD patients to undertake a gluten challenge. Whole blood assessment coupled with ultra-sensitive cytokine detection shows promise in the monitoring of rare antigen-specific T cells in clinical studies.

## Introduction

Antigen-specific CD4+ T cells are drivers of both inflammatory and tolerogenic immune responses. Functional blood-based biomarkers for antigen-specific CD4+ T cells play a central role in characterizing immune responses, and in monitoring vaccines and therapies intended to induce or suppress antigen-specific immunity, or restore immune tolerance (1). Autoimmune diseases including coeliac disease (CD) can often be associated with significant target organ injury, but in contrast to acute infectious diseases, conventional functional assays are often not sufficiently sensitive to detect relevant peripheral blood antigen-specific CD4+ T cells (2, 3).

Cytokine release assays (CRAs) employing fresh or cryopreserved peripheral blood mononuclear cells (PBMC) have been the mainstay for studying and monitoring T-cell immunity in patients (4). A practical challenge for immune monitoring in clinical trials is to preserve the functional properties of CD4+ T cells, which requires that PBMC should be separated from whole blood within 24 hours before cryopreservation or commencing an assay (5). CRAs using fresh, unseparated blood added directly to antigen soon after blood collection have previously been evaluated as research tools (6), and are now used for diagnosis of infectious diseases such as tuberculosis (7). Whole blood is typically “stimulated” by addition of an activator of innate or adaptive immunity, and maintained at 37°C in a sealed tube or dispensed into 96-well microplates and placed in an incubator. Plasma is separated after completing usually six to 48-hours incubation, frozen, and transported to a central laboratory for specialized biomarker assay. Newer versions of whole blood CRAs have been promoted for profiling cytokine release to various stimuli (8, 9), but in our past experience whole blood CRA has been lacking the sensitivity to detect cytokine release by rare gluten-specific CD4+ T cells in CD patients (10).

CD has many features in common with autoimmune disease (11). CD is strongly associated with HLA-DQ2.5, and CD4+ T cells specific for a well-documented hierarchy of gluten epitopes play a central role in intestinal injury, and are drivers of acute gluten-induced cytokine release and symptoms (12, 13). Gluten-specific CD4+ T cells isolated from intestinal tissue or in PBMC share many of the same surface markers suggesting blood is a valid source of gluten-specific CD4+ T cells to monitor gluten immunity (14).

Only two methods have consistently identified rare peripheral blood gluten-specific CD4+ T cells in CD patients. Blood is either collected 6 to 8 days after subjecting patients to a short gluten-food challenge to increase their frequencies allowing detection by conventional CRAs (10, 15–17), or PBMC are stained with customized MHC class II-gluten peptide tetramers, concentrated by immunomagnetic beads, and then enumerated by flow cytometry (18). These experimental approaches have shown that the frequency of peripheral blood gluten-specific CD4+ T cells is below ten per million CD4+ T cells in treated CD, but increases about ten-fold by six days after commencing gluten challenge (17, 19). Recently, in parallel to the current study, we first reported a simple whole blood CRA utilizing an electrochemiluminescence (ECL) immunoassay for interleukin-2 (IL-2) that appears to be as effective as MHC class II-gluten peptide tetramers for detection of these rare gluten-specific CD4+ T cells in CD, and can be combined with multiplex assessments of interferon-γ (IFN-γ) and interleukin-10 (IL-10) (3). Based on Poisson distribution analysis, we detected approximately 0.5 – 11 IL-2 secreting gluten-specific T cells per one mL of fresh whole blood collected from treated CD patients without gluten challenge.

In the present study, we utilize this novel CRA for rare gluten-specific CD4+ T cells with the aim of optimizing blood collection and processing to monitor the effects of an experimental peptide immunotherapy (Nexvax2) for CD during a phase 2 clinical trial.

## Materials and Methods

### Study Design

**Figure 1:**
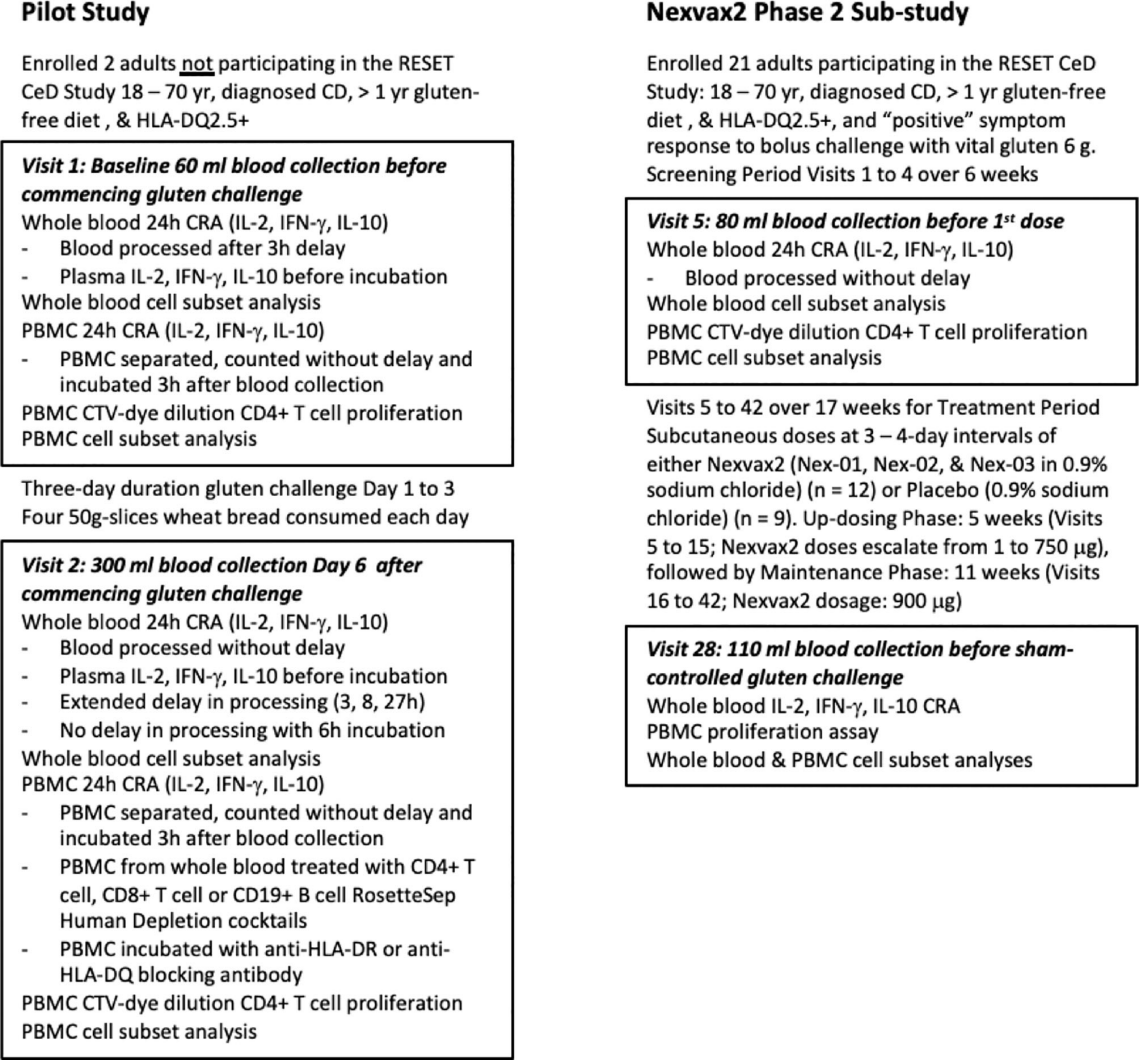
Study overview. The pilot study included two participants prior to commencement of the Nexvax2 Phase 2 Sub-study.

Immune monitoring sub-studies supplementing the RESET CeD Study are outlined in **Figure 1**. Nexvax2 is an experimental antigen-specific immunotherapy intended to restore clinical and immunological tolerance to gluten in HLA-DQ2.5+ patients with CD. The “RESET CeD Study” was a double-blind, placebo-controlled phase 2 efficacy study of Nexvax2 conducted at 39 trial sites and randomized a total of 178 HLA-DQ2.5+ adults with treated, medically confirmed CD (ClinicalTrials.gov identifier: NCT03644069) (20). The primary endpoint in the RESET CeD Study was self-reported gastrointestinal symptoms the day of a masked single bolus gluten challenge after 14-weeks treatment compared to a baseline period pre-treatment. Nexvax2 was administered in aqueous 0.9% sodium chloride twice weekly as a subcutaneous injection, which results in detectable plasma levels of each constituent peptide over about six hours (21). The bioactive component of Nexvax2 is limited to the three gluten-related peptides shown in **Table 1** (Nex-01, Nex-02, and Nex-03) that each includes immuno-dominant HLA-DQ2.5-restricted epitopes for gluten-specific CD4+ T cells (22).

**Table 1:** Peptides and their final concentrations in PBMC and whole blood incubations^†^.

Peptide	Source	Length^‡^	Amino acid sequence (Z pyroglutamate)	Solvent	ControlPool	NXPool	xNXPool	NXxNXPool	CEFTPool
Nex-01	α-gliadin/Nexvax2	16	ZLQPFPQPELPYPQPQ-NH_2_	PBS		5 μM		5 μM	
Nex-02	ω-gliadin/Nexvax2	15	ZQPFPQPEQPFPWQP-NH_2_	PBS		5 μM		5 μM	
Nex-03	B-hordein/Nexvax2	16	ZPEQPIPEQPQPYPQQ-NH_2_	PBS		5 μM		5 μM	
W04	ω-gliadin	16	ZPFPQPEQPIPVQPEQ-NH_2_	PBS			5 μM	5 μM	
R03	ω-gliadin	16	ZPFPQPEQPTPIQPEQ-NH_2_	PBS			5 μM	5 μM	
W14	ω-secalin	16	ZIQPEQPFPEQPEQIR-NH_2_	PBS			5 μM	5 μM	
SCP1	Scramble	8	ZPFPLPQP-NH_2_	PBS	15 μM	7.5 μM	7.5 μM		15 μM
SCP2	Scramble	8	ZPQYQPEQ-NH_2_	PBS	15 μM	7.5 μM	7.5 μM		15 μM
SCP3	Scramble	8	ZPFEPQPL-NH_2_	PBS	15 μM	7.5 μM	7.5 μM		15 μM
SCP4	Scramble	8	ZPQSYPEQ-NH_2_	PBS	15 μM	7.5 μM	7.5 μM		15 μM
CEFT	CMV, influenza, EBV, & C. tetani	9 - 21	27 peptides with HLA class I or II restricted T-cell epitopes	DMSO					0.1 μg/ml

^†^225 μl unseparated, heparinized blood was dispensed into wells containing 25 μl PBS with 10x final concentration of peptides;

^‡^Number of amino acids.

A convenient blood test that did not require patients to be rechallenged with gluten was needed for serial assessments of rare CD4+ T cells specific for epitopes represented in Nex-01, Nex-02, or Nex-03, and for other gluten-specific CD4+ T cells that may be indirectly suppressed by Nexvax2 immunotherapy through induction of regulatory T cells (23, 24). Initially, in a pilot study separate from the RESET CeD Study, PBMC and whole blood were compared as sources of gluten-specific CD4+ T cells for the CRA. Subsequently, the CRA using whole blood was deployed to monitor Nexvax2 immunotherapy during the RESET CeD Study in a subgroup of CD patients enrolled at three sites in Melbourne, Australia. Additional blood was collected before treatment (Visit 5), during maintenance treatment (Visit 28), and at the end of treatment (Visit 42). However, after an interim analysis indicated the primary efficacy endpoint in the RESET CeD study would not be achieved, dosing was discontinued on 25 June 2019 and the supplementary sub-study was also halted. Consequently, sample analysis for the supplementary sub-study was limited to Visit 5 and 28.

### Participants and Ethics Approvals

The pilot study was approved by the Human Research Ethics Committees at the Walter and Eliza Hall Institute and Melbourne Health (identifiers 03/4 and 2003.009 respectively), and the RESET CeD study was approved by Melbourne Health Human Research Ethics Committee (Study Number HREC/43048/MH-2018) and Bellberry Limited (Application Number 2018-07-562-A-13). Patients for the pilot study were recruited at The Royal Melbourne Hospital and the Walter and Eliza Hall Institute of Medical Research (Parkville, VIC). The two volunteers in the pilot study did not participate in the RESET CeD Study. Patients for the sub-study in the RESET CeD study were recruited from The Royal Melbourne Hospital/Walter and Eliza Hall Institute of Medical Research (n = 19), Box Hill Hospital (n = 2), and Alfred Hospital (n = 2). All patients enrolled at these sites were invited to volunteer and did enroll in the sub-study. All patients gave written, informed consent prior to undergoing any study procedures. Full eligibility criteria for the RESET CeD are published elsewhere (20). All participants in the RESET CeD Study were aged between 18 and 70 years, had a diagnosis of CD made on the basis of duodenal histology showing villous atrophy and supportive serology, had been on a gluten free diet (GFD) for at least one year, were HLA-DQ2.5 positive, and had worsening of gastrointestinal symptoms within six hours after consuming 10-grams vital wheat gluten as described elsewhere (20).

### Clinical Procedures

Interventions for the pilot study included a 3-day gluten challenge and blood collections at baseline (60 ml) and one week later on Day-6 after commencing gluten challenge (300 ml). Blood was collected as previously described *via* 21G ¾“ Surflo winged infusion set (Terumo, Shibuya City, Japan) into 10 ml lithium-heparin vacutainers (Becton Dickinson, Franklin Lakes, New Jersey, USA) (25). Gluten challenge for the pilot study comprised four 50-gram slices of gluten-containing wheat bread consumed daily for three consecutive days as previously described (15). For the supplementary sub-study of the RESET CeD Study, heparinised blood (80 ml) was collected at Visit 5 just before patients received their first dose of Nexvax2 or placebo, and again at Visit 28 (110 ml) after patients had received 12-weeks treatment with two-times weekly subcutaneous injections of study drug. The dosing schedule for Nexvax2 included eleven escalating doses from 1 μg to 750 μg described elsewhere (21), which was followed by maintenance dosing at 900 μg. Visit 28 marked the first of three masked single bolus food challenges that are described elsewhere and were gluten-free or contained 10-grams vital wheat gluten (20). Most patients in the supplementary sub-study had been discontinued from the RESET CeD Study before completing masked single bolus gluten challenge at Visit 34 or Visit 42 at the end-of-treatment. Consequently, immune monitoring was limited to blood collected at Visit 5 and Visit 28.

### Peptides and Antigens

**Table 1** shows the individual peptides and peptide pools used in the study. CS Bio (Menlo Park, CA, USA) synthesized each of the peptides except for CEFT (JPT Peptide Technologies, Berlin, Germany). Immunogenic gluten peptides sequences were selected from the hierarchy of gluten peptides determined by IFN-γ ELISpot using PBMC from blood collected from treated HLA-DQ2.5+ CD patients six days after they commenced gluten challenge (26). Peptide purity was > 95% by HPLC, and LC-MS confirmed each peptide’s identity. As described elsewhere (3), peptide pool stock solutions were prepared as 10x final incubation concentration and were dispensed in 25 μl to individual wells in sterile 96-well U-bottom microwell plates (Thermo Fisher Scientific) that would later be used for blood incubations. Plates were sealed with sterile adhesive covers (Thermo Fisher Scientific), stored at -80°C, and shipped on dry ice to the study site. Immediately before use, to avoid condensation and seepage of peptide solutions between wells, microplates containing test solutions were thawed while being centrifuged at room temperature.

### PBMC Separation

Heparinized blood was diluted with an equal volume of PBS/2% fetal bovine serum (FBS) (StemCell Technologies) before transfer to 50 ml SepMate™ tubes (StemCell Technologies) pre-filled with 15 ml Ficoll-Paque Plus (GE Healthcare). Blood was separated by centrifugation at 1200g for 10 min at room temperature. The majority of the plasma supernatant was removed and discarded. The remainder of the layer above the SepMate barrier was poured into a fresh tube and washed twice in PBS/2% FBS, centrifuge settings: 300g for 10 min at room temperature. PBMC were counted using the Scepter cytometer (Merck) with 40 μm sensors. PBMC were resuspended to 0.9-1.8 million per ml in RPMI 1640 media supplemented with 10% Human male AB serum (Sigma-Aldrich), 1x Glutamax (Gibco), 1x MEM non-essential amino acids (Gibco) and 50 μM 2-mercaptoethanol (Sigma-Aldrich).

### Cytokine Release Assay

The CRA was performed as described elsewhere (3). Briefly, 225 μl aliquots of blood or PBMC in media were dispensed into wells pre-filled with 25 μl PBS containing “NX”, “xNX”, “NXxNX”, “Control”, or “CEFT” peptides (**Table 1**). Six replicate wells were assessed for each test condition. Incubation plates were immediately placed in a humidified incubator at 37°C in 5% CO_2_. After the incubation period, plates were centrifuged at 500g for 10 min at room temperature. Plasma, 90 - 120 μl per well, was carefully collected to avoid blood cell contamination and transferred to a corresponding well in a “mirror image” sterile 96-well plate that was sealed with an adhesive cover, immediately frozen at -80°C, and later shipped to the ImmusanT laboratory for cytokine assessment. An ECL 3-plex IL-2, IFN-γ, and IL-10 immunoassay kit from Meso Scale Diagnostics LLC (Rockville, MD, USA) was used according to manufacturer’s instructions. In the pilot study, one 25 µL plasma sample was assessed from each of six replicate incubations. In the RESET CeD Study, plasmas from two adjacent replicate wells were pooled making three measurements for each test condition. Cytokine concentration was determined using the MSD MESO™ Sector S600 plate reader and Discovery Workbench 4.0. The lower limit of detection (LLOD) was calculated for each cytokine on each assay plate.

### HLA-DQ and HLA-DR Blocking

After resuspending PBMC to 0.9 - 1.8 million per ml in RPMI/10% Human AB serum, two 2 ml fractions were transferred to fresh tubes and incubated with anti-HLA-DQ antibody at 10 μg/ml (clone SPVL3; Beckman Coulter) or anti-HLA-DR antibody at 10 μg/ml (clone L243; BioLegend) for one hour at 37°C. Test conditions were assessed in duplicate wells.

### Cell Depletions

Whole blood was depleted of either CD4+ T cells, CD8+ T cells or CD19+ B cells using RosetteSep Human Depletion cocktails and SepMate tubes (Stemcell Technologies), according to manufacturer’s recommendations. Briefly, heparinized blood was divided between 3 tubes and incubated for 10 mins at room temperature with either CD4 Depletion Cocktail, CD8 Depletion Cocktail or a custom CD19 Depletion Cocktail (all at 1:20 dilution). The whole blood was then diluted and loaded into SepMate tubes and PBMC were isolated according to the procedure described above. The unwanted cells pellet with the red blood cells and therefore are depleted from the resulting PBMC. The depleted PBMC were resuspended at 0.9 - 1.8 million per ml in complete RPMI/10% Human AB serum, and then incubated with peptides in the cytokine release assay as described above. Test conditions were assessed with duplicate wells.

### Peptide-Stimulated Proliferation of CD4+ T Cells

As described previously (3), proliferation assays consisted of three replicate wells per condition containing ~0.3 million in 225 μl per well of CTV-labelled PBMC and 25 μl test solution. After 8 days cells were stained with CD3-FITC (UCHT1), CD4-APC (SK3), and 7-Amino-Actinomycin D to discriminate dead cells (7-AAD; all from BD Biosciences). Cells were analysed on a BD FACSVerse cytometer and flow cytometry data was analysed by FlowJo software (version 10; FlowJo, LLC).

### Lymphocyte Subset and Monocyte Frequencies in Fresh Blood and PBMC

Whole blood or PBMC were stained with antibody mix comprising anti-human CD3-Bv421, CD4-PE, CD8-APC, CD14-APCH7, CD19-Bv480, CD20-PECy7, and CD45-FITC (clones UCHT1, SK3, SK1, MphiP9, SJ25C1, L27, 2D1, respectively) and 7-AAD (all from BD Biosciences). For whole blood, BD Trucount™ tubes were used according to the manufacturer’s instructions (**Figure S1**). Briefly, antibody mix was added followed by 50 μl whole blood by reverse pipetting. Tubes were vortexed and incubated for 15 min at room temperature in the dark. Erythrocytes were lysed by incubating with 450 μl 1xPharm Lyse™ (BD Biosciences), for 15min before analysing samples on a BD FACS Verse.

### Statistics

The sample size was empirical for both the pilot and exploratory mechanistic study. All participants in the RESET CeD Study who had samples collected at Visit 5 and 28 were included in the analysis. Cytokine release (pg/ml) was normalized by dividing by the number of CD4+CD3+ T cells per well, or, to account for HLA class I restricted epitopes in CEFT and CD8+ T cell depletion studies, by the sum of CD4+CD3+ and CD8+CD3+ T cells per well. Non-parametric statistical tests were used to compare unpaired (Mann-Whitney U test) or paired data (Wilcoxon signed-rank test) without correction for multiple comparisons. ANOVA test was used to compare changes in biomarkers associated with treatment in the RESET CeD Study. For statistical tests, cytokine concentrations for signal values below the LLOD were treated as equal to the LLOD.

## Results

### Exploratory Functional Assessments of Peripheral Blood Gluten-Specific CD4+ T Cells

The two CD patients (S0568 and S0211) in the pilot study each provided blood samples before and six days after commencing 3-day gluten challenge to assess CRAs and CD4+ T cell proliferation stimulated by gluten peptides (**Figure 1**). They were studied from 3 December to 11 December 2018. Both participants were HLA-DQ2.5+ and heterozygous for HLA-DQA1*05 and HLA-DQB1*02. S0211 was a male aged 67 years on GFD for 20 years, and S0568 was a 62-year-old female on GFD for 17 years.

### Gluten Peptide-Stimulated CD4+ T Cell Proliferation in PBMC

**Figure 2:**
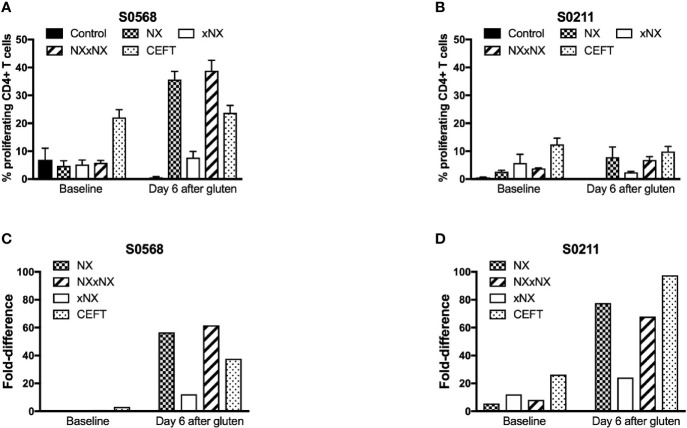
CD4+ T cell proliferation assessed by CTV-dilution in fresh PBMC from two HLA-DQ2.5+ CD patients incubated for 8 days with peptide pools containing Nexvax2 peptides (NX), non Nexvax2 peptides (xNX) or both, or CEFT positive control peptides. Mean (SEM) percent proliferating CD4+ T cells **(A, B)** and mean fold-difference in proliferation compared to control **(C, D)** is shown for PBMC incubated with peptide mixes, data represent mean of triplicates.

Dye-dilution proliferation assays are well described for detection of rare antigen-specific CD4+ T cells (27, 28). PBMC from blood collected before and six days after gluten challenge were used in 8-day CTV dye-dilution proliferation assays. For PBMC assessed after patients had had a 3-day gluten challenge, CD4+ T-cells proliferated in response to each gluten peptide pool (NX, xNX and NXxNX), and the CEFT peptide pool (**Figures 2A–D**). For S0211, the fold-difference for NX compared to the control scrambled 8mer peptide pool was 5.7 before versus 77 after gluten challenge. In contrast, S0568’s responses to NX and other gluten peptide pools were no different from control before gluten challenge, but after gluten challenge the NX-stimulated response was 57-times higher than control. Altogether, these findings indicated that the CTV dye-dilution proliferation assay was suitable for detection of gluten-specific CD4+ T cell responses, but may not be sufficiently sensitive in some CD patients without prior gluten challenge.

### Gluten Peptide-Stimulated CRA in PBMC

**Figure 3:**
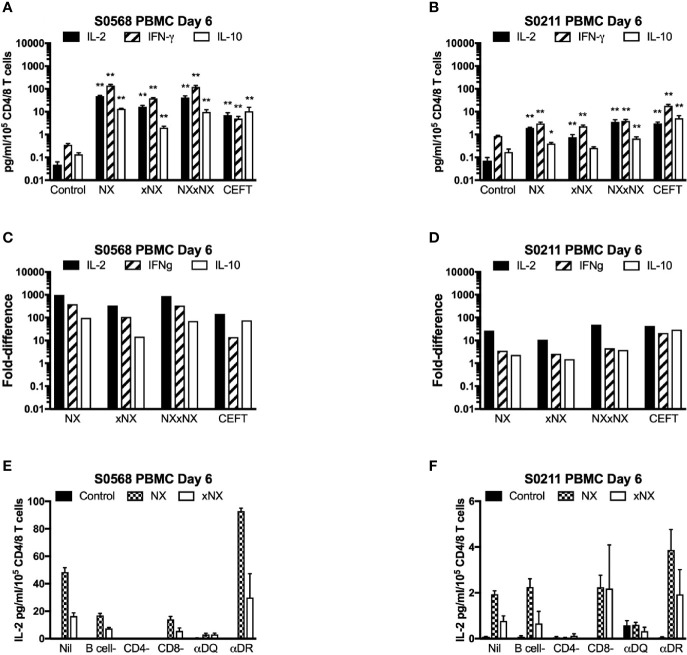
Cytokine release in fresh PBMC from two HLA-DQ2.5+ CD patients. Concentrations **(A, B)** and fold-change relative to control **(C, D)** are shown for IL-2, IFN-γ, and IL-10 in media after 24-hour incubation of PBMC with gluten peptides or CEFT. Data are normalized for the number of live CD3+CD4+ and CD3+CD8+ T lymphocytes in PBMC and represent the mean + SEM of six replicate wells; Mann-Whitney U test was used to compare cytokine release for peptide mixes versus Control (statistical significance is indicated by *p < 0.05, or **p < 0.01). IL-2 release in PBMC is shown following depletion of B cells, CD4+ T cells or CD8+ T cells, or pre- and co-incubation with antibody that blocks epitope presentation by HLA-DQ or HLA-DR, data represent mean of duplicates **(E, F)**.

**Figure 4:**
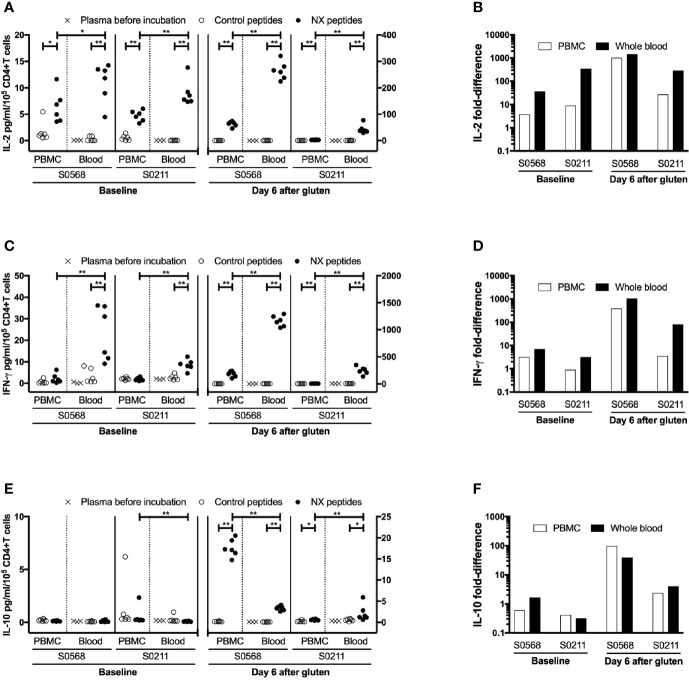
Concentration and fold-difference relative to control for Nexvax2 (NX)-stimulated IL-2 **(A**, **B)**, IFN-γ **(C**, **D)** and IL-10 **(E**, **F)** release in PBMC and whole blood normalised for the number of live CD3+CD4+ T cells. Unseparated blood was allowed to stand at room temperature while PBMC were separated from a matched sample. Three hours after blood was collected, 225 μl aliquots of whole blood or 0.5 million PBMC in media were dispensed into microwells containing 25 ul PBS and test antigen. Plasma or media were separated after 24-hour incubation. For whole blood incubations, cytokine concentrations in plasma separated from whole blood before incubation with NX or control is also shown. Data points represent cytokine levels for each of six replicates assessed for each condition with blood collected before and six days after commencing gluten challenge for two coeliac disease patients. Mann-Whitney U test was used to compare NX-stimulated cytokine release in PBMC versus fresh whole blood; statistical significance is indicated by *p < 0.05, or **p < 0.01.

With the aim of developing a simple but sensitive assay of gluten-specific CD4+ T cells, a 24-hour PBMC-based CRA using ECL to assess IL-2, IFN-γ, and IL-10 release was tested using six replicates for each condition with PBMC from blood collected after gluten challenge. Both patients showed significantly increased IL-2, IFN-γ, and IL-10 release stimulated by NX, xNX and NXxNX, and CEFT peptide pools compared to the negative control pool (**Figures 3A, B**). Overall, IL-2, IFN-γ and IL-10 release stimulated by NXxNX was always significantly higher than xNX (p < 0.05), but was significantly higher than NX only for IL-2 and IL-10 release by S0211 (p < 0.05) and not for any cytokine tested in S0568. Expressed as a ratio to the negative control, IL-2 release for gluten peptide pools (NX, xNX and NXxNX) were on average 2.8- or 7.5-times higher than for IFN-γ, and 15- or 10-times higher than for IL-10 in S0568 and S0211, respectively (**Figures 3C, D**). CRAs with PBMC separated from blood that had CD4+ or CD8+ T cells, or B cells depleted indicated that CD4+ T cells but not CD8+ T cells or B cells were necessary for NX and xNX-stimulated IL-2 release (**Figures 3E, F**). NX and xNX-stimulated IL-2 secretion was also reduced by incubating PBMC with anti-HLA-DQ antibody, but not by anti-HLA-DR antibody (**Figures 3E, F**). However, when the PBMC-based CRA was applied to cells isolated from blood collected before gluten challenge, only IL-2 release was significantly increased in PBMC stimulated with gluten peptide pools (**Figures 4A, B**), and neither IFN-γ or IL-10 release were detected (**Figures 4C–F**).

Collectively, these observations indicated this ECL-based CRA was capable of detecting IL-2, IFN-γ and IL-10 release in PBMC stimulated by gluten peptides. But without prior gluten challenge this CRA using PBMC could only detect relatively weak gluten peptide-stimulated IL-2-release. This fell short of the suggested minimal measure of T-cell function for clinical monitoring that should include both IL-2 and IFN-γ secretion (4).

### Cytokine Release Stimulated by Gluten-Peptides in Whole Blood Compared to PBMC

We speculated that cytokine release in whole blood may be more efficient than using PBMC because of reduced delays and less manipulation of immune cells prior to stimulation with antigen. A portion of the heparinized blood collected from the two CD patients before and after gluten challenge was allowed to stand undisturbed at room temperature in the collection tube while the remainder was used to prepare PBMC. After PBMC were separated, the frequencies of CD4+CD3+ T cells, CD8+CD3+ T cells, CD19+CD20+ B cells, and CD14+ monocytes were assessed in PBMC and whole blood. After 3-hours, PBMC had been separated, resuspended in media, and cell density had been adjusted so that the number of CD4+ T cells per 225 μl (median: 0.19 million, range: 0.17 - 0.21 in a total of 0.5 million PBMC) was similar to the expected number of CD4+ T cells in 225 μl of matched, unseparated blood (median: 0.16 million, range: 0.13 - 0.21) (**Table 2**). This ensured that the immune cell population in each well incubated with gluten peptides was matched for the frequencies of responding CD4+ T cells and antigen presenting cells, and for delay since blood collection.

**Table 2:** Cytokine concentrations in plasma or media^†^, assay dynamic range, and cell subset frequencies in matched 24-hour whole blood and PBMC cytokine release assays.

Patient		S0568				S0211			
Sample		Baseline		Day 6 after gluten		Baseline		Day 6 after gluten	
Matrix		Whole blood	PBMC	Whole blood	PBMC	Whole blood	PBMC	Whole blood	PBMC
**Pre-incubation**									
Cell frequencies per well	CD3+CD4+	128500	169000	205000	205500	182000	181000	130000	214500
	CD3+CD8+	56500	78500	76500	75000	100000	119500	69500	112000
	CD19+CD20+	48500	33000	62500	41500	60000	21500	34000	28500
	CD14+	32500	87000	52500	55000	35500	73000	37000	53000
IL-2	Plasma/media	ND	<LLOD	<LLOD	<LLOD	<LLOD	<LLOD	<LLOD	<LLOD
pg/ml	LLOD	0.09	0.09	0.09	0.09	0.09	0.09	0.09	0.09
IFN-γ	Plasma/media	<LLOD	ND	1.11	<LLOD	3.64	<LLOD	5.7	<LLOD
pg/ml	LLOD	0.88	0.88	0.88	0.88	0.88	0.88	0.88	0.88
IL-10	Plasma/media	0.12	ND	0.231	<LLOD	0.308	<LLOD	0.41	<LLOD
pg/ml	LLOD	0.04	0.04	0.04	0.04	0.04	0.04	0.04	0.04
**Post-incubation with peptides mixes**									
IL-2	Control	0.39	2.9	0.37	0.13	0.05	0.94	0.19	0.23
pg/ml	NX	14	11	543	130	16	8.5	57	6.2
	xNX	3.0	4.1	154	44	7.7	3.8	30	2.5
	NXxNX	12	13	574	113	13	9.3	48	7.6
	CEFT	185	37	136	19	89	35	84	9.9
	LLOD	0.04	0.04	0.04	0.04	0.05	0.05	0.06	0.06
	ULOD	2940	2940	2940	2940	2940	2940	2940	2940
IFN-γ	Control	4.3	1.2	2.23	0.96	5.0	3.9	4.0	2.8
pg/ml	NX	30	3.8	2378	374	16	3.5	318	9.8
	xNX	11	2.9	591	103	7.7	3.4	129	7.2
	NXxNX	14	4.2	2307	328	13	3.0	178	13
	CEFT	362	37	245	14	366	181	264	59
	LLOD	0.49	0.49	0.49	0.49	0.49	0.49	0.47	0.47
	ULOD	2860	2860	2860	2860	2860	2860	2860	2860
IL-10	Control	0.09	0.34	0.17	0.37	0.51	2.5	0.71	0.55
pg/ml	NX	0.15	0.21	6.9	36	0.16	1.0	2.9	1.3
	xNX	0.16	0.22	2.2	5.4	0.44	1.3	2.3	0.84
	NXxNX	0.03	0.22	7.4	26	0.10	0.51	1.3	1.8
	CEFT	0.79	5.0	2.0	28	2.8	9.3	0.95	17
	LLOD	0.02	0.02	0.03	0.03	0.03	0.03	0.02	0.02
	ULOD	680	680	680	680	680	680	680	680

^†^Mean of six replicate wells except for pre-incubation plasma/media that were assessed in triplicate.

LLOD, lower limit of detection; ULOD, upper limit of detection.

Before the 24-hour incubation with peptide mixtures, IL-2 concentrations were consistently below the lower level of detection, LLOD (0.04 pg/ml) in plasma from whole blood, whereas median IFN-γ and IL-10 concentrations were 2.4 and 0.27 pg/ml, respectively (**Table 2**). As expected, none of the three cytokines were detectable in media used to resuspend PBMC. After 24-hour incubation with control peptides, median IL-2, IFN-γ and IL-10 concentrations measured in plasma from the two whole blood samples collected from both CD donors (total of four blood samples studied) had modestly increased to 0.28, 4.2, 0.34 pg/ml, respectively, which were similar to their concentrations in media after 24-hour incubations of matched PBMC (0.59, 2.0, and 0.46 pg/ml, respectively) (**Table 2**). After normalizing for the frequency of live CD4+ T cells present in each well when incubation commenced, IL-2 release stimulated by NX relative to control was consistently higher in whole blood than matched PBMC incubations (p < 0.05) with median IL-2 release 10-times (range: 1.5 - 39) higher for whole blood than PBMC (**Figures 4A, B**). IFN-γ release stimulated by NX relative to control was also consistently higher in whole blood than matched PBMC incubations (p < 0.01) with median IFN-γ release 3.1-times (range: 2.1 - 23) higher for whole blood (**Figures 4C, D**). In contrast, IL-10 release was not consistently changed, and in two of the four blood samples, IL-10 release was significantly lower in whole blood than PBMC (**Figures 4E, F**). Other gluten peptide pools and CEFT also showed substantially greater IL-2 and IFN-γ release relative to control in whole blood than in matched PBMC (**Table 2**).

**Figure 5:**
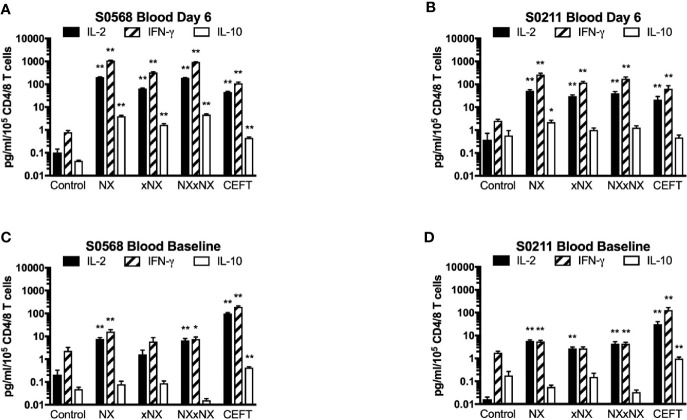
Gluten peptide-stimulated 24-hour fresh whole blood IL-2, IFN-γ, and IL-10 release normalised for the number of live CD3+CD4+ and CD3+CD8+ T cells in individual microwells were compared to the negative control in samples from two HLA-DQ2.5+ CD patients: after gluten challenge **(A**, **B)** or at baseline before gluten challenge **(C**, **D)**. Bars represent the mean and SEM of six replicate wells. Mann-Whitney U test was used to compare gluten peptide-stimulated cytokine release versus Control; statistical significance is indicated by *p < 0.05, or **p < 0.01.

In addition to cytokine secretion in whole blood being more robust than with PBMC after gluten challenge, whole blood at baseline before gluten challenge also showed significantly increased IFN-γ as well as IL-2 release stimulated by NX, NXxNX and CEFT compared to control for both CD patients (**Figure 5**). At baseline, whole blood IL-2 release stimulated by NX was 37- and 351-times higher than control for S0568 and S0211, respectively, whereas whole blood IFN-γ release stimulated by NX was 16- and 3.1-times higher than control, respectively. However, none of the peptide pools except CEFT stimulated IL-10 release in whole blood at baseline. Whole blood IL-2 and IFN-γ release stimulated by NX at baseline were consistently significantly higher than for xNX (p < 0.05), but usually not significantly different from NXxNX.

Collectively, these findings indicated that using whole blood instead of PBMC substantially enhanced cytokine secretion stimulated by antigenic peptides, and allowed the ECL-based CRA to measure gluten peptide-stimulated IL-2 and IFN-γ release without requiring CD patients to undertake a gluten challenge.

### Effects of Duration of Whole Blood Incubation on Cytokine Release

**Figure 6:**
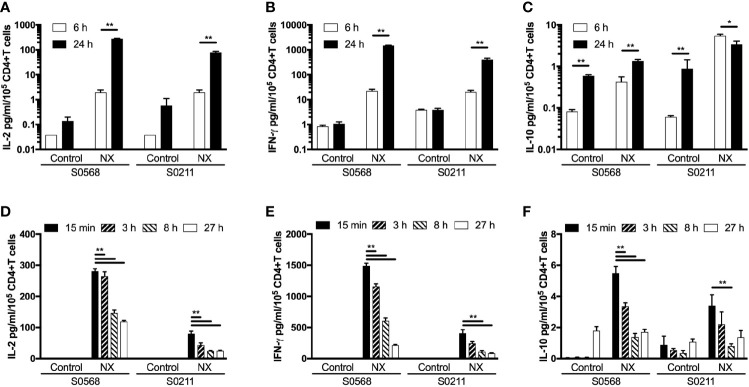
Shortening the incubation period **(A**–**C)**, and varying the period blood was kept in collection tubes at room temperature **(D**–**F)** were assessed for their effects on gluten peptide-stimulated fresh whole blood IL-2, IFN-γ, and IL-10 release. Blood samples from two HLA-DQ2.5+ CD patients collected after gluten challenge were studied. Bars represent the mean and SEM of six replicate wells. Statistical significance by Mann-Whitney U test *p < 0.05, **p < 0.01.

The kinetics of tetanus toxoid-stimulated whole blood cytokine release indicates that IL-2, IFN-γ, and IL-10 release begin only after six hours, and peak concentrations of IL-2, IFN-γ and IL-10 are reached at or after 24 hours, but assessments with longer incubations may be compromised by hemolysis (6). CRAs with whole blood plated into 96-well microplates without delay were used to test whether CRAs would be informative with incubations reduced to six hours. Blood incubated with NX for 24 hours compared to six-hours had 141- or 40-times higher plasma concentrations of IL-2, and 66- or 20-times higher plasma concentrations of IFN-γ for S0569 and S0211, respectively (**Figures 6A–C**). There were no significant differences between six- and 24-hour IL-2 or IFN-γ release for control. These findings indicated gluten peptide-stimulated whole blood IL-2 and IFN-γ release largely occurred after six hours, which is notably slower than IL-2 release *in vivo* after CD patients consume gluten (13).

### Effects of Delaying Incubation of Whole Blood on Cytokine Release

Delays of up to 24-hours before processing blood for functional T-cell assays are considered acceptable in multi-centre immunotherapy clinical trials, but validation studies have often utilized PBMC that may have been cryopreserved (5). To assess the effects of delaying incubation of whole blood with antigen, blood from both CD patients on day 6 after commencing gluten challenge was collected into 10 ml lithium-heparin vacutainers that remained capped at room temperature for three, eight or 27 hours before addition to 96 well microplates and commencing 24-hour incubation. Blood that was processed without delay was dispensed directly into microplates and commenced incubation with antigen within 25 minutes of collection. Frequencies of live cells for each immune cell subset were assessed in blood samples immediately before incubations commenced. Compared to blood processed without delay, the average NX-stimulated IL-2 release normalized for the frequency of living CD4+ T cells declined significantly to 74% after three hours delay, to 41% at eight hours, and to 37% by 27 hours (**Figure 6D**). Normalized NX-stimulated IFN-γ release also dropped significantly to 69% after three hours delay, to 34% at eight hours, and to 18% by 27 hours (**Figure 6E**). A similar reduction in NX-stimulated IL-10 release was also observed (**Figure 6F**). **Table S1** shows that the decline in IL-2, IFN-γ and IL-10 release with increasing delay in commencing incubation was similar for NX, xNX, NXxNX and CEFT.

These findings indicated that delays of more than three hours between collection and incubation of fresh blood with antigen were likely to alter the functional phenotype of immune cells and substantially reduce antigen-stimulated cytokine secretion.

### Monitoring Gluten Peptide-Stimulated T-Cells During Nexvax2 Immunotherapy

There were 23 participants in the RESET CeD study who enrolled in the supplementary sub-study from 20 December 2018 to 24 July 2019, but two were excluded as they did not have blood collected at both Visit 5 and 28. Demographics of the 12 participants who received Nexvax2 and 9 who received placebo treatment was similar, except that five among those receiving Nexvax2 were HLA-DQB1*02 homozygotes compared to one receiving placebo (**Table 3**).

**Table 3:** Demographics and characteristics of RESET CeD Study participants.

Treatment	Nexvax2 (n=12)	Placebo (n=9)
Mean (SD) age in years	45 (11)	49 (16)
Number (%) females	8 (67)	4 (44)
Mean (SD) height in centimeters	171 (12)	176 (9)
Mean (SD) body mass in kilograms	81 (16)	80 (14)
Mean (SD) body mass index	28 (5)	26 (4)
Number (%) Caucasian	12 (100)	9 (100)
Number (%) positive for anti-TG2 IgA or DGP IgG	2 (17)	0 (0)
Number (%) positive for HLA-DQ2.5	12 (100)	9 (100)
Number (%) homozygous for HLA-DQB1*02	5 (42)	1 (11)
Secretory IgA deficiency	1 (8)	0 (0)
Median (range) years age at diagnosis CD	39 (18 - 54)	36 (22 - 61)
Median (range) years following GFD	8 (1 - 20)	10 (6 - 18)
Median (range) peak GloSS^†^ after screening gluten challenge	8 (4 - 9)	8 (3 - 10)
Median (range) serum IL-2 4 h after screening gluten challenge (pg/ml)	6.3 (<0.5 - 36)	3.2 (<0.5 - 81)

^†^GloSS is self-rated “global gastrointestinal symptom score” rated from no symptoms (0) to very severe (10) during the 6 hours after bolus food challenge with 10 g vital wheat gluten at screening.

**Figure 7:**
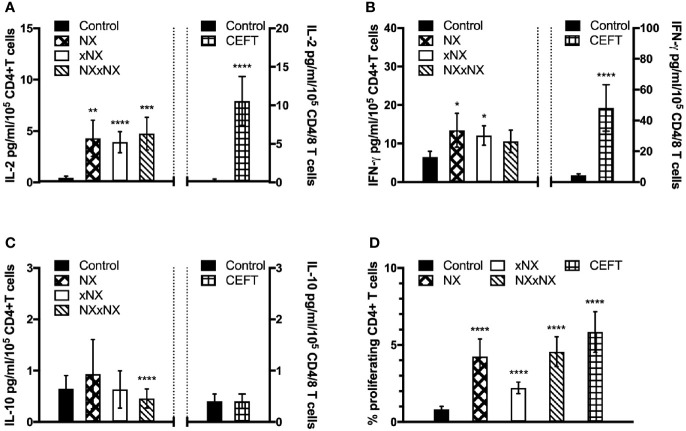
Gluten-specific T cell responses prior to Nexvax2 administration. Gluten peptide-stimulated whole blood CRA **(A–C)** and CD4+ T cell proliferation measured by 8-day CTV-dye dilution assay before treatment **(D)** at Visit 5 in 21 patients randomized in the RESET CeD Study. Bars represent the mean and SEM. Wilcoxon signed-rank test was used to compare gluten peptide-stimulated cytokine release versus Control; statistical significance is indicated by *p < 0.05, **p < 0.01, ***p < 0.001, or ****p < 0.0001.

**Figure 8:**
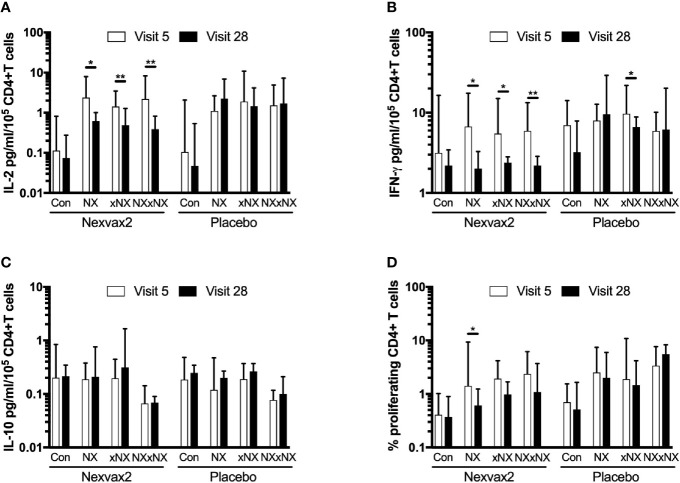
Change in gluten peptide pool-stimulated whole blood CRA **(A–C)** and CD4+ T cell proliferation measured by 8-day CTV-dye dilution assay **(D)** over 12 weeks from before treatment at Visit 5 to Visit 28 during maintenance treatment in patients treated with Nexvax2 (n = 12) or placebo (n = 9) in the RESET CeD Study. Medians and interquartile ranges are indicated. Wilcoxon signed-rank test was used to compare Visit 5 to Visit 28; statistical significance is indicated by *p < 0.05, or **p < 0.01.

The ECL-based 24-hour whole blood IL-2, IFN-γ and IL-10 release assay normalized for the frequency of CD4+ T cells, and the 8-day CTV-dye dilution assay for CD4+ T-cell proliferation assay were used to monitor the effects of Nexvax2 immunotherapy. Overall, the median delay between blood collection and commencing incubation for CRA was 40 min (range: 10 to 270 min), and the median difference between the delay at Visit 5 and Visit 28 for individual patients was 0 min (range: -50 to +95 min). At Visit 5 (n = 21), compared to the negative control, there were statistically significant increases in IL-2 and IFN-γ release, and CD4+ T cell proliferation stimulated by NX, NXxNX, and CEFT, and for IL-2 release and CD4+ T cell proliferation stimulated by xNX (**Figure 7**). Comparing Visit 5 and Visit 28 showed statistically significant reductions in NX-stimulated whole blood IL-2 and IFN-γ release, and CD4+ T cell proliferation in Nexvax2-treated patients, but not in placebo-treated patients (**Figure 8**, **Table S2**). In addition, xNX- and NXxNX-stimulated whole blood IL-2 and IFN-γ release fell significantly between Visit 5 and Visit 28 in Nexvax2-treated patients, albeit there was also a significant but isolated fall in xNX-stimulated IFN-γ release in the placebo group. Comparing the differences in biomarker measurements between Visit 5 and Visit 28 for Nexvax2 treatment compared to placebo, there were trends for reduction in NX- and NXxNX-stimulated IL-2 (p = 0.056, and p = 0.056, respectively by ANOVA test) and for NX-stimulated IFN-γ release (p = 0.0802, ANOVA test). Overall, Nexvax2 therapy appeared to have reduced gluten peptide-stimulated peripheral blood IFN-γ release so that it was no different from control, attenuated gluten peptide-stimulated peripheral blood IL-2 release and CD4+ T cell proliferation, but did not enhance gluten peptide-stimulated peripheral blood IL-10 release.

## Discussion

In the present study and in a companion report (3), we used the MSD VPLEX multiplex ECL immunoassay for IL-2, IFN-γ and IL-10 to develop a highly sensitive whole blood CRA for rare peripheral blood gluten-specific CD4+ T cells. Here we demonstrate that standard practices for handling and processing blood can profoundly suppress antigen-stimulated IL-2 and IFN-γ release, which compromises detection of rare gluten-specific CD4+ T cells. The new whole blood CRA along with a dye-dilution proliferation assay were effective tools for monitoring gluten immunity in CD patients without needing a gluten food challenge to expand the pool of gluten-specific CD4+ T cells. These innovations allowed us to show that gluten peptide-stimulated whole blood IL-2 and IFN-γ release, and CD4+ T cell proliferation were significantly reduced in CD patients during treatment with Nexvax2 peptide immunotherapy, but not in placebo-treated CD patients. Interestingly, Nexvax2 therapy was associated with reduced ex vivo responses to gluten peptides present in Nexvax2 as well as an expanded pool of gluten peptides, which could support a mechanism of action involving direct suppression of Nexvax2-specific CD4+ T cells as well as indirect suppression of gluten-reactive CD4+ T cells not specific for epitopes in Nexvax2.

Previously, we and others have relied on 3-day gluten challenge to mobilize gluten-reactive CD4+ T cells in blood to monitor gluten-reactive CD4+ T cells by functional assays (22). Avoiding the need for gluten challenges in the immune monitoring sub-study associated with the RESET CeD Study was critical because patients had to comply with the RESET CeD Study protocol requiring no additional gluten exposures apart from the per-protocol masked gluten challenges required for the primary and secondary endpoints. PBMC had initially been preferred for CRAs because they could also be cryopreserved for later testing of CD4+ T cell function, staining with MHC class II-gluten peptide tetramers for flow cytometry assessments of surface phenotype, gene expression and DNA-based assays including T cell receptor gene analysis. However, finding that gluten peptides stimulated no detectable IFN-γ or IL-10 release, and only modest IL-2 release in PBMC without prior gluten challenge of donors prompted a detailed assessment of gluten peptide-stimulated cytokine release in whole blood.

IL-2 and IFN-γ release stimulated by gluten peptide pools and CEFT were normalized for the total numbers of CD4+ T cells or CD4+ and CD8+ T cells per well, respectively, which allowed direct comparisons between matched incubations of PBMC and whole blood. IL-2 and IFN-γ release stimulated by gluten peptide pools and CEFT in PBMC were typically one-tenth and one-third, respectively, the amounts measured using whole blood. The cause for reduced cytokine secretion by antigen-activated T cells in PBMC is unclear, but could relate to injury of reactive T cells due to physical manipulation, effects of Ficoll, or media replacing plasma. Further investigations are required in larger numbers of blood samples to confirm these unexpected findings. Our observations could explain why CRAs using PBMC have been unable to detect rare antigen-specific CD4+ T cells, and position whole blood CRAs using high sensitivity immunoassays as an attractive new tool to study and monitor rare antigen-specific CD4+ T cells. In addition to the detrimental effects of separating PBMC from whole blood, this study also showed IL-2 and IFN-γ release stimulated by gluten peptide pools and CEFT were substantially reduced by delaying incubation of whole blood with antigen. Gluten peptide-stimulated IL-2 and IFN-γ release in blood allowed to stand for 24 hours at room temperature were reduced to one-third and one-fifth, respectively, the amount in blood that was incubated within 30 minutes of collection. This observation also challenges current practice in multicenter clinical trials that allows blood for functional T-cell assays to be processed at a central laboratory within 24 hours after collection.

Our finding that gluten peptide-stimulated whole blood IL-2 release is more pronounced than IFN-γ, and also IL-10 release, and is a sensitive marker for rare gluten-specific CD4+ T cells is in keeping with our parallel study that profiled cytokine release in CD patients when blood was incubated with a single immunodominant α-gliadin peptide identical to NEX-01 (3). In that study, we showed that the hierarchy of cytokines and chemokines released in whole blood stimulated by the α-gliadin peptide was IL-2 followed by IFN-γ, CXCL10/IP-10, CXCL9/MIG, IL-10, CCL3/MIP-1α, and TNF-α, in that order. We also showed that the whole blood assay was superior to other functional readouts of the antigen-specific T cell response such as the IFN-γ ELISpot, which is unable to detect gluten-specific T cell responses in treated CD patients without gluten challenge. The sensitivity of the whole blood IL-2 release assay appeared to be similar to MHC class II-gluten peptide tetramers, which have indicated the frequency of CD4+ T cells specific for epitopes in Nexvax2 is usually less than 10 per million peripheral blood CD4+ T cells in CD patients without prior gluten challenge (17, 19). A volume of 40 to 50 ml of blood is required for a single assessment by MHC class II-gluten peptide tetramers specific for epitopes in Nexax2 gluten peptides (17, 18), which contrasts with the whole blood CRA that detected Nexvax2 gluten peptide-stimulated IL-2 and IFN-γ release in 1.35 ml blood divided across six replicate wells each containing about 0.2 million CD4+ T cells. Therefore, in this study we estimate that an average total of about 12 gluten-specific CD4+ T cells distributed across six replicate wells was sufficient to monitor IL-2 and IFN-γ release stimulated by the pool of gluten peptides included in Nexvax2.

Finally, application of the whole blood CRA to monitor gluten-specific CD4+ T cells in the RESET CeD Study revealed that Nexvax2 treatment was associated with attenuation of gluten peptide-stimulated IL-2 and IFN-γ release, and CD4+ T cell proliferation. Statistical comparisons between the twelve Nexvax2 and nine placebo-treated patients in this sub-study almost reached significance for the reduction of IL-2 and IFN-γ release stimulated by gluten peptides in Nexvax2, and IL-2 release stimulated by the expanded pool of gluten peptides. This outcome was also unexpected, and may indicate that relatively small cohorts are needed to achieve statistical significance using whole blood CRAs to monitor antigen-specific immunotherapies in early clinical development. An explanation remains to be found for the discrepancy between significant reductions in ex vivo gluten peptide-stimulated IL-2 and IFN-γ release in whole blood, and the primary efficacy endpoint of the RESET CeD Study not being met. Self-reported digestive symptoms within one day after masked single-bolus gluten challenge compared to the pretreatment period was the primary endpoint. Patients ingested 10 grams of vital wheat gluten for the masked gluten challenge. This gluten challenge resulted in almost half of the placebo-treated patients in the RESET CeD Study experiencing vomiting between one to two hours, and was associated with median 20-fold increase of serum IL-2 at four hours (20). Serum IL-2 elevations correlated with nausea severity and vomiting (20), but the relationships between gluten exposure, symptoms, intestinal T cell activation, mucosal cytokine release, and systemic measures of gluten immunity including serum cytokines and responsiveness of peripheral blood gluten-specific CD4+ T cells remain to be determined. Nonetheless, the dose and/or duration of Nexvax2 administration was inadequate to suppress symptoms triggered by a food challenge administering almost half the usual daily gluten intake in an unrestricted diet as a single bolus.

A limitation of this work is that only two volunteers were assessed in the pilot study. However, findings from these two patients were consistent and demonstrated the capacity of the whole blood assay approach to detect gluten-specific T cells prior to a gluten challenge. Subsequent validation in the cohort of subjects assessed from the RESET CeD study strongly supported the sensitivity of the whole blood assay approach without gluten challenge. As gluten peptide-stimulated whole blood IL-2 and IFN-γ release was shown to be strongly affected by experimental conditions such as delayed incubation and overall incubation time, further work is required to understand the conditions supporting optimal assay performance. Future work should correlate whole blood IL-2 and IFN-γ release with assessment of PBMC by HLA-DQ2.5-gluten-peptide tetramers and flow cytometry in CD patients who have not been gluten challenged.

The whole blood CRA coupled with ultra-sensitive IL-2 and IFN-γ detection has promise as an immunomonitoring tool for clinical trials. In order to scale the protocol for multi-centre clinical trials, further refinements to address the challenges of blood transport delay from clinical sites distant from the laboratory will be important. For example, antigen-coated vacutainers could be employed for direct in-tube incubation of blood rather than multi-well plates, similar to that used in QuantiFERON-TB Gold *Mycobacterium tuberculosis* diagnostic kits, or the antigen could be added straight to blood in the collection tubes (22). This would expedite sample incubation at each trial site. Optimization of the antigens may also be useful.

In conclusion, rare gluten-specific CD4+ T cells can be detected and monitored using a simple whole blood CRA that utilizes a sensitive ECL immunoassay to measure IL-2 and IFN-γ release. Sensitivity of CRAs to detect peripheral blood gluten-specific CD4+ T cells is markedly compromised by utilizing PBMC instead of whole blood, and by delays in incubating blood with antigen. Deploying this whole blood CRA to monitor effects of Nexvax2 immunotherapy was highly informative. This simple protocol in an optimized form may allow widespread utilization of this test to monitor the function of rare antigen-specific CD4+ T cells in clinical trials assessing vaccines and immunotherapy.

## Data Availability Statement

The original contributions presented in the study are included in the article/**Supplementary Material**. Further inquiries can be directed to the corresponding author.

## Ethics Statement

The studies involving human participants were reviewed and approved by the Human Research Ethics Committees at the Walter and Eliza Hall Institute of Medical Research, Melbourne Health, and Bellberry Limited. The patients/participants provided their written informed consent to participate in this study.

## Author Contributions

RA, GG, LW, JD, and JT-D designed the studies. JT-D, SC, GB, KG, KN, and KT conducted clinical studies. MH, AR, SW, RZ, ES, and JD performed and analyzed immune assays. RA and GG provided data integration and analysis. RA wrote the manuscript and prepared the Tables and Figures. RA had full access to all the data in the study and had final responsibility for the decision to submit for publication. All authors contributed to the article and approved the submitted version.

## Funding

Funding for this work was provided by ImmusanT, Inc., Cambridge, Massachusetts USA. JT-D and his team (AR, MH) were supported by the NHMRC (APP1176553), the University of Melbourne (Mathison Centenary Fellowship), the Beck Family Foundation and Coeliac Australia.

## Conflict of Interest

ImmusanT, Inc., Cambridge, Massachusetts USA provided funding for the study. ImmusanT was involved in the study design, collection of data, and the decision to submit it for publication. RA, GG, SW, ES, RZ, KG, KN, KT, LW, and JD were employees of ImmusanT, Inc. JT-D has served as an advisor to ImmusanT, Inc. RA and JT-D are inventors of Patents owned or licensed by ImmusanT, Inc. that relate to gluten peptide immunotherapy and diagnostics.

The remaining authors declare that the research was conducted in the absence of any commercial or financial relationships that could be construed as a potential conflict of interest.

## Supplementary Material

Supplementary Figure 1 Cell subset FACS gating strategy for whole blood cell counts. 50 μl of whole blood was stained in TruCOUNT tubes and the number of each subset per μl of whole blood was calculated using the manufacturer’s provided equation: (Number of events in region containing cells of interest/Number of events in absolute count bead region) × (Number of beads per test as per the TruCOUNT tube foil pouch/test volume in μl). This was used to determine cell numbers per well.

## Abbreviations

CRA, cytokine release assay; CD, coeliac disease; CTV, CellTrace™ Violet dye; DGP, deamidated gliadin peptide; DMSO, dimethyl sulphoxide; ECL, electrochemiluminescence; GC, gluten food challenge; ELISpot, enzyme-linked immune absorbent spot; GFD, gluten-free diet; HLA-DQ, Human Leukocyte Antigen-DQ; HLA-DR, Human Leukocyte Antigen-DR; Ig, immunoglobulin; IL-2, interleukin-2; IFN-γ, interferon-γ; LLOD, lower limit of detection; PBMC, peripheral blood mononuclear cells; PBS, phosphate buffered saline; SFU, Spot-forming unit; TG2, transglutaminase 2; ULOD, upper limit of detection.
