# Supplementary figures and images for: A Sensitive Whole Blood Assay Detects Antigen-Stimulated Cytokine Release From CD4+ T Cells and Facilitates Immunomonitoring in a Phase 2 Clinical Trial of Nexvax2 in Coeliac Disease
Source: Front Immunol. 2021 May 19;12:661622. doi: 10.3389/fimmu.2021.661622 (PMC8171185; doi:10.3389/fimmu.2021.661622)

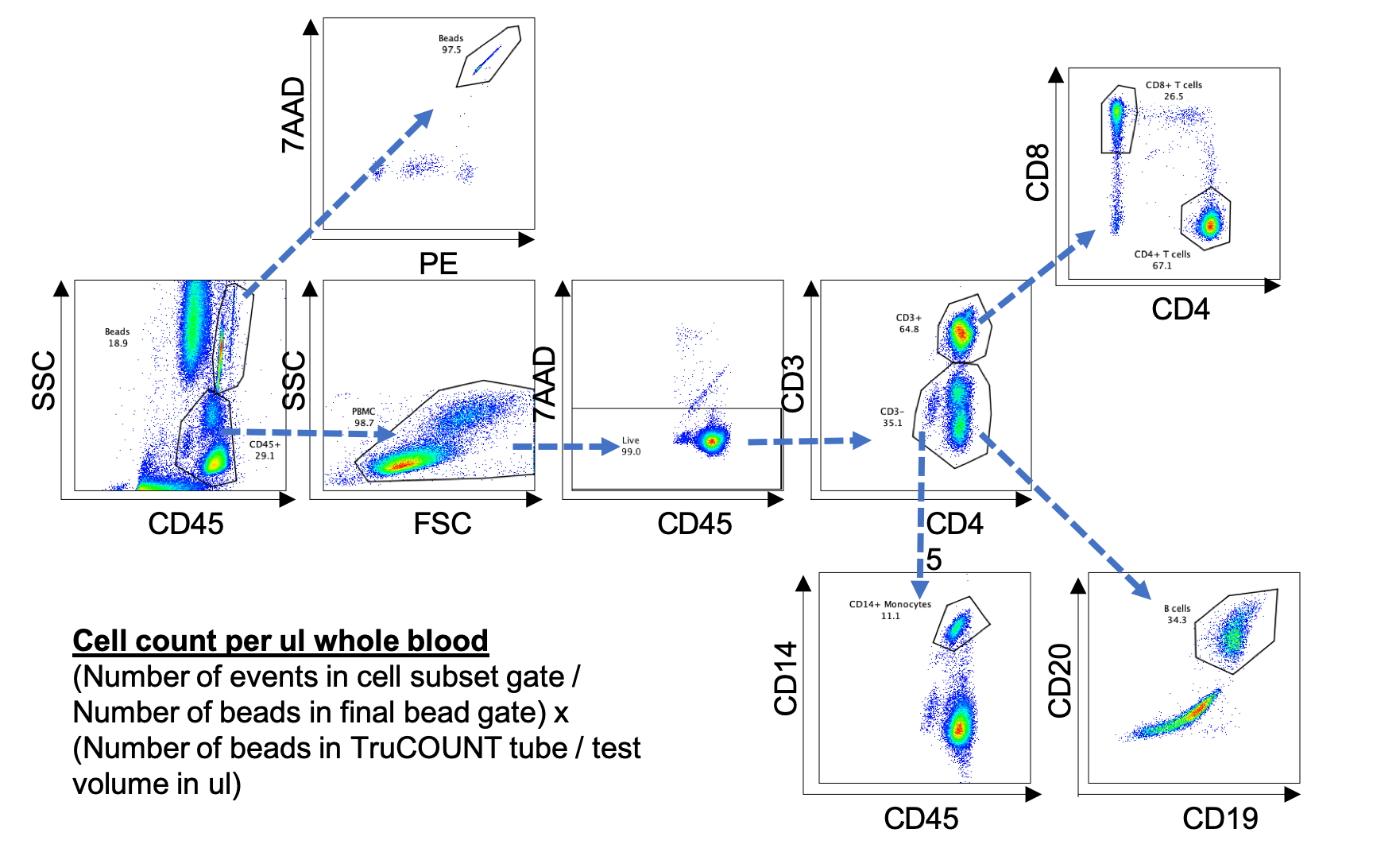

Supplement: Supplementary Figure 1 — Cell subset FACS gating strategy for whole blood cell counts. 50 μl of whole blood was stained in TruCOUNT tubes and the number of each subset per μl of whole blood was calculated using the manufacturer’s provided equation: (Number of events in region containing cells of interest/Number of events in absolute count bead region) × (Number of beads per test as per the TruCOUNT tube foil pouch/test volume in μl). This was used to determine cell numbers per well. [file Image_1.png]
